# A framework for pathway knowledge driven prioritization in genome‐wide association studies

**DOI:** 10.1002/gepi.22345

**Published:** 2020-08-10

**Authors:** Shrayashi Biswas, Soumen Pal, Partha P. Majumder, Samsiddhi Bhattacharjee

**Affiliations:** ^1^ National Institute of Biomedical Genomics Kalyani India

**Keywords:** GWAS, LASSO penalty, logistic regression, pathways, prioritization, weighted *p* values

## Abstract

Many variants with low frequencies or with low to modest effects likely remain unidentified in genome‐wide association studies (GWAS) because of stringent genome‐wide thresholds for detection. To improve the power of detection, variant prioritization based on their functional annotations and epigenetic landmarks has been used successfully. Here, we propose a novel method of prioritization of a GWAS by exploiting gene‐level knowledge (e.g., annotations to pathways and ontologies) and show that it further improves power. Often, disease associated variants are found near genes that are coinvolved in specific biological pathways relevant to disease process. Utilization of this knowledge to conduct a prioritized scan increases the power to detect loci that map to genes clustered in a few specific pathways. We have developed a computationally scalable framework based on penalized logistic regression (termed *GKnowMTest*—*G*enomic *Know*ledge‐guided *M*ultiplte *Test*ing) to enable a prioritized pathway‐guided GWAS scan with a very large number of gene‐level annotations. We demonstrate that the proposed strategy improves overall power and maintains the Type 1 error globally. Our method works on genome‐wide summary level data and a user‐specified list of pathways (e.g., those extracted from large pathway databases without reference to biology of a specific disease). It automatically reweights the input *p* values by incorporating the pathway enrichments as “adaptively learned” from the data using a cross‐validation technique to avoid overfitting. We used whole‐genome simulations and some publicly available GWAS data sets to illustrate the application of our method. The *GKnowMTest* framework has been implemented as a user‐friendly open‐source R package.

## 1 INTRODUCTION

Genome‐wide association studies (GWAS) have discovered thousands of single nucleotide polymorphisms (SNPs) robustly associated with various common complex diseases and traits (Visscher et al., 2017). However, many common variants that are truly associated with common diseases possibly still remain to be detected above the stringent genome‐wide significance threshold (e.g., $5\times 10^{-8}$). To discover all such variants by standard GWAS (or meta‐anlaysis) alone would require huge sample sizes (Zhang, Qi, Park, & Chatterjee, 2018). Individually many of these SNPs would have modest effects but collectively they can explain a significant percentage of missing heritability of the diseases/traits (Speed, Cai, Johnson, Nejentsev, & Balding, 2017) and also help in enhancing the biological understanding about genes and pathways involved in pathogenesis. Hence, it is crucial to develop complementary approaches to accelerate the discovery of common disease‐associated variants from GWAS data. With this realization, there has been substantial and consistent interest in developing statistical methods for prioritizing SNPs in a GWAS based on prior knowledge, such as their location, functional annotations, epigenetic features or expression quantitative trait loci (eQTLs) evidence (Iversen, Lipton, Clyde, & Monteiro, 2014; Nicolae et al., 2010; Pickrell, 2014; Schork et al., 2013; Sveinbjornsson et al., 2016; Yang, Fritsche, Zhou, & Abecasis, 2017).

In the context of prioritizing SNPs in GWA studies, the primary focus has been on SNP‐level annotations such as those based on physical context of a SNP (genic/intergenic or exonic/intronic) and tissue specific functional annotations overlapping a SNP (e.g., promoter/enhancer marks, open chromatin, etc.), or SNPs assoicated with multiple correlated traits (Bhattacharjee et al., 2012; Ellinghaus et al., 2016). Lesser attention has been given to gene‐level annotations (such as pathways), in terms of using pathway knowledge a priori to guide discovery in GWAS. Pathways constitute a rich source of prior knowledge in the sense that, the genes mapping to loci identified from GWAS of particular disease tend to cluster into a few specific biological pathways/networks. This prior information is ignored when one conducts an unbiased traditional GWAS. To give a hypothetical example, suppose that 90% of all infectious disease susceptibility SNPs map to genes within a small number of pathways (say k pathways) related to immunity. Due to accumulation of several small effects of multiple variants, such pathways can be identified as “relatively important” empirically based on a “peek” at the GWAS data. A SNP “A” on a gene that maps to k′ (<k) of these out of these k important pathways is a priori much more likely to be a true susceptibility SNP, compared to another SNP “B” that is in a gene desert or a SNP “C” that maps to genes in other pathways, and hence it is natural to upweight SNP “A” relative to SNPs “B” and “C” as a genome‐wide multiple‐testing strategy to improve overall power of discovery. In this strategy, multiple SNPs clustering in some common pathways boost each other to receive higher weight, whereas SNPs in isolated or insufficiently annotated genes automatically get down‐weighted.

It is important to note that prespecifying a set of “important pathways” based on subjective knowledge can lead to huge power loss when the candidate pathways turn out to be incorrect (Roeder, Devlin, & Wasserman, 2007). Ideally the relative importance of pathways should be automatically determined from the GWAS data. Given GWAS results, it is possible to map SNPs onto genes and then conduct pathway enrichment analysis (Wang, Li, & Hakonarson, 2010) or network analysis (Jia & Zhao, 2014) to biologically interpret findings. However, such post hoc enrichment analysis does not lead to discovery of additional (novel) SNPs beyond those with *p* values below genome‐wide significance threshold ($p<5\times 10^{-08}$). For this, we need a pathway‐guided GWAS strategy that can help discover more SNPs by improving the statistical power of detection. Some existing methods allow incorporation of pathways in GWAS analysis using Bayesian models to achieve prioritization and return posteriors or Bayes factors as output (e.g., Bush, Dudek, & Ritchie, 2009; Carbonetto & Stephens, 2013; Chen & Thomas, 2010; Lee, Blom, Wang, Shim, & Marcotte, 2011). Here, we develop a generic framework for pathway‐based prioritization that allows the user to adhere to the usual *p* value threshold for genome‐wide significance. Pathway‐guided GWAS can be particularly useful for secondary reweighted analysis of GWAS data for making additional discoveries. The method is specifically designed to be able to incorporate knowledge from a large number of pathways in a scalable computationally efficient manner. Further, it is easy to implement in any statistical software package making it accessible for routine use by the community.

The proposed method starts by accepting summary‐level data (e.g., *p* values of each SNP from a GWAS) and a user‐supplied list of pathways as input. It maps the SNPs onto genes (based on physical location) and then onto the pathways. The “prior” chance of each SNP to be “truly” associated, is estimated in a data‐driven manner based on the “degree of enrichment” of the pathway(s) that the SNP maps to. Once the priors are determined, the multiple testing penalty is allocated differentially so that SNPs with higher prior are penalized less and have greater chance of being discovered. Here, we used the *p* value weighting method (Roeder & Wasserman, 2009; Roeder et al., 2007) that allowed us to maintain the overall false‐positive rate at the same stringency as a traditional GWAS (e.g., $p<5\times 10^{-8}$) and at the same time weights can be allocated “optimally” in the sense of maximizing overall power of discovery. The method returns weighted *p* values of each SNP which can be treated as *p* values from a standard GWAS in the sense of global error and a standard Manhattan plot can be used to visualize results.

To achieve computational scalability, we use a novel technique termed PMLR (*P*osterior *M*arginal *L*ogistic *R*egression) to fit our model and estimate the prior probability of each SNP based on annotations. This approach only requires fitting penalized logistic regression for model fitting, making it fast and stable in terms of convergence even in presence of large number of annotations. Through genome‐wide simulations we demonstrate that our approach maintains correct global false‐positive rates and improves power. We demonstrate application of the method on multiple real GWAS datasets (e.g., psoriasis, type 2 diabetes). The framework has been implemented in an R package “GKnowMTest” (*G*enomic *Know*ledge‐guided *M*ultiple *Test*ing) that is freely available from *github*. It will be submitted to the R/Bioconductor repository that conveniently hosts a rich collection of annotation packages and mirrors some publicly available pathway databases and ontologies.

## 2 METHODS

### 2.1 Statistical method and algorithms

Our algorithm consists of two distinct modules for (a) “Enrichment‐Estimation” of annotation categories, where the degree of enrichment of each functional annotation is estimated from the genome‐wide data (e.g., summary‐level *Z*‐scores) and (b) “Prioritized‐Testing” step where Type 1 error is allocated differentially to the annotation categories based on the enrichments. For each module we propose efficient and computationally scalable methods as described below.

#### 2.1.1 Model and notation

Suppose we have summary‐level data in the form of *Z*‐scores $Z_{1},Z_{2},\text{…},Z_{M}$ for each of the M SNPs tested in a GWAS. Further, we have K user‐specified categories of SNPs (henceforth called “annotations”) defined based on knowledge external to the GWAS. Specifically, here we consider gene‐level priors in the form of user‐specified “gene sets” (pathways). SNPs are mapped to pathways based on their physical proximity to genes of each pathway (see section “Mapping SNPs to gene‐level annotations”). Thus each SNP is mapped either to a few pathways or remains unmapped. This provides an annotation matrix for the SNPs, $V_{jk}=1$ if the *j*th SNP maps to the *k*th annotation and $V_{jk}=0$ otherwise.

We assume that the *Z*‐scores of each SNP are marginally drawn from a two‐group mixture model of a null (N(0,1)) and an alternate distribution $f_{1}$, with the “prior probability of being a true SNP” $\pi_{j}$ possibly varying across SNPs. We assume that this prior probability is determined by the annotations of the SNP through a logistic linear model. Thus, the *Z*‐score of the *j*th SNP is distributed as follows:

(1) $$\begin{array}{c} Z_{j}\sim \left\{\begin{array}{cc} N\left(0,1\right) & \delta_{j}=0;\\ f_{1} & \delta_{j}=1. \end{array}\right)\\ \text{and}\;\delta_{j}\sim \mathrm{Ber}\left(\pi_{j}\right),\;\text{with}\\ \log\frac{\pi_{j}}{1-\pi_{j}}=\gamma +V_{j}\eta , \end{array}$$

where γ denotes the log‐odds of a SNP to be associated if it does not belong to any of the annotations and η denotes the vector of log‐odds ratios contributed by each annotation. Logistic modeling is commonly used to accommodate large number of prior annotations (Iversen et al., 2014; Pickrell, 2014). However a crucial difference in our scenario is that, the $\delta_{j}=1$ in our model refers to an associated SNP (for univariate GWAS), whereas in other contexts it indicates causality of a SNP in a multivariate model, which is meaningful in the context of identifying causal variants.

#### 2.1.2 Enrichment estimation by PMLR

The product of marginal likelihoods of each SNP can be maximized to obtain reasonably good estimates (see “Supporting Information Methods”) of the parameters. It is given as product of the mixture density for each SNP that is

$$\begin{array}{ccc} L\left(\gamma ,\eta \right) & = & \overset{M}{\underset{j=1}{\prod }}\left\{\pi_{j}f_{1}\left(z_{j}\right)+\left(1-\pi_{j}\right)N\left(z_{j};0,1\right)\right\}\\ & = & \overset{M}{\underset{j=1}{\prod }}\left\{\frac{\text{exp}\left(\gamma +V_{j}\prime \eta \right)}{1+\text{exp}\left(\gamma +V_{j}\prime \eta \right)}f_{1}\left(z_{j}\right)+\frac{1}{1+\text{exp}\left(\gamma +V_{j}\prime \eta \right)}N\left(z_{j};0,1\right)\right\} \end{array}$$

Maximization of the likelihood model can be computationally difficult when there are a large number of annotations, even more so in presence of penalization (e.g., LASSO penalty). We propose a novel method to estimate the model coefficients termed PMLR involving two sequential steps.

##### Obtaining posterior marginals

As the first step, the prior information is ignored and marginal “Posterior Probability of Association” (mPPA) of each SNP is derived. This can be done in various ways such as by assuming $f_{1}$ to be normal (Carbonetto & Stephens, 2013; Kichaev et al., 2014; Pickrell, 2014; Roeder & Wasserman, 2009; Roeder et al., 2007). Here we use a more nonparametric approach following the local FDR method of Efron (2005). Efron's local FDR works by obtaining the nonparametric density estimate of the marginal distribution $\hat{f}$ of the *Z*‐scores. The local‐fdr for the *j*th SNP is then given by

(2) $$lfdr\left(j\right)=Pr\left(\delta =0\;|\;z_{j}\right)=\frac{N\left(0,1;z_{j}\right)\hat{\pi_{0}}}{\hat{f}\left(z_{j}\right)},$$

where $\hat{\pi_{0}}$ and $\hat{f}$ denote, respectively, the estimated “proportion of null SNPs” and “marginal density.” The mPPA of each SNP is then given by $\psi_{j}=1-lfdr\left(j\right)$.

##### Penalized logistic regression to derive priors

To derive SNP prior probabilities, we use penalized logistic regression of the mPPA values on the annotations and a sparseness penalty as follows. When there are a large number of SNPs having identical set of annotations the SNPs can be merged into an equivalence class and represented as a single row. The average within‐class mPPA value is used as the response. This strategy can provide substantial computational savings (see “Supporting Information Methods”).

(3) $$\log\frac{\bar{\psi_{i}}}{1-\bar{\psi_{i}}}=\kappa +\beta \prime V_{i}$$

where $V_{i}$ denotes the *i*th row of the annotation matrix V, corresponding to the *i*th equivalence class. To fit the parameters we minimize the function below for specific values of α and λ, that is,

(4) $$\min_{\kappa ,\beta }-2*loglik\left(\kappa ,\beta \right)+\lambda \left\{\alpha \sum \left|\beta_{k}\right|+\left(1-\alpha \right)\sum \beta_{k}^{2}\right\},$$

where loglik(κ,β) denotes the log‐likelihood function based on the logistic model. For model fitting, see sections “Computation and Scalability” and “Choice of Tuning Parameter” below. Finally, the shrunk estimates of the SNP prior probabilities are given by the fitted values.

(5) $$\hat{\pi_{i}}=\frac{exp\;\left(\hat{\kappa }+\beta \prime V_{i}\right)}{1+exp\;\left(\hat{\kappa }+\beta \prime V_{i}\right)}.$$

The above procedure can be interpreted as a single step of an EM‐algorithm starting from a valid initial estimate (as shown in the “Supporting Information Methods”).

#### 2.1.3 Prioritized multiple testing

After prior probabilites are estimated, existing methods control FDR or posterior probabilities of SNPs or regions to be true (Carbonetto & Stephens, 2013; Kichaev et al., 2014; Pickrell, 2014). Recently (Sveinbjornsson et al., 2016) proposed different genome‐wide thresholds for SNPs based on annotations under global FWER control that is analogous to weighting of *p* values (Roeder & Wasserman, 2009). Traditionally, in the context of GWAS, family‐wise error rate (FWER) has been the accepted standard for Type 1 error control. Hence we restrict to the FWER‐controlling weighted *p* value approach in this study, so that the conventional criterion for GWAS significance (*p* < 5e−08) can be used after the reweighting of *p* values.

Given prior probabilities of truths $\left(\hat{\pi_{i}}\right)$ in each annotation group, we use a decision theoretic criterion similar to Roeder and Wasserman (2009). We consider the maximizing the expected number of true positives among all the tests constraining the expected number of false‐positives (under the global null) to the desired FWER level (α=.05).

Assuming $w_{i}\prime$ are the SNP‐level *p* value weights, the expected number of true positives is

(6) $$E\left(TP\right)=h\left(w_{1}^{\prime },w_{2}^{\prime },\text{…},w_{C}^{\prime }\right)=\sum_{i=1}^{C}n_{i}\;\hat{\pi_{i}}\;\bar{F_{1}}\left\{{\bar{\Phi }}^{-1}\left[\frac{\alpha w_{i}^{\prime }}{M}\right]\right\},$$

where $\bar{F_{1}}$ and $\bar{\Phi }$ denote the null and alternative upper tail CDFs of the *Z*‐scores. This needs to be minimized under the gobal Type 1 error constraint $\bar{w\prime }=1$ (see “Supporting Information Methods” for details). The weighted *p* values discussed above are not uniformly distributed under the null hypothesis. However, they can be treated as usual *p* values in the sense that the global (average) Type 1 error is maintained at the target level α.

##### Cubic *p* value weights (CPW)

Here, we assume the SNP‐level weights in a class (at the log‐scale) to be a cubic function of the prior log‐odds of truth for SNPs in that class, that is

$$w_{i}\prime =exp\left(\gamma_{0}+\gamma_{1}\beta_{i}+\gamma_{2}\beta_{i}^{2}+\gamma_{3}\beta_{i}^{3}\right),$$

where $\beta_{i}=\log\left[\frac{\hat{\pi_{i}}}{1-\hat{\pi_{i}}}\right]$. We performed limited experiments with higher order polynomials, but did not find any significant improvement after cubic exponent. Invoking the constraint that $\bar{w\prime }=1$, we get,

$$w_{i}\prime =\frac{exp\left(\gamma_{1}\beta_{i}+\gamma_{2}\beta_{i}^{2}+\gamma_{3}\beta_{i}^{3}\right)}{\sum_{i}n_{i}exp\left(\gamma_{1}\beta_{i}+\gamma_{2}\beta_{i}^{2}+\gamma_{3}\beta_{i}^{3}\right)∕M}$$

Finally, power (i.e., E[TP]) as in Equation (6) can be maximized over weights parametrized by $\left(\gamma_{1},\gamma_{2},\gamma_{3}\right)$, an unconstrained optimization problem. As an alternative distribution we used $N\left(\mu ,1+\tau^{2}\right)$ here, but a nonparametric CDF estimate $\hat{F_{1}}$ can be used instead.

##### Simple *p* value weights (SPW)

This scheme is essentially CPW with $\left(\gamma_{1}=1,\gamma_{2}=0,\gamma_{3}=0\right)$. This is similar to simple weights “proportional to odds‐ratio” considered previously (Sveinbjornsson et al., 2016). However, it should be noted that, in our context, odds of a category is based on coefficients of multiple regression rather than an univariate analysis.

#### 2.1.4 Mapping SNPs to gene‐level annotations

To incorporate pathway knowledge into GWAS it is first required to map SNPs onto pathways. For this, we obtained the physical positions of the SNPs from dbSNP and that of the genes listed in the input pathway using a reference map of transcripts based on the same genome build (e.g., hg19). Any SNP mapping to any of the genes in a particular pathway was considered to map to that pathway/annotation (See “Supporting Inforamation Methods”). We used biological knowledge from three different databases. These were (a) the gene lists for 229 KEGG pathways (Kanehisa & Goto, 2000), (b) 142 gene sets comprising transcription factors and their validated targets derived from Transfac (Matys et al., 2006) and (c) 2,326 Gene‐Ontology (Biological Processes; Ashburner et al., 2000) by merging or breaking the nodes with too few or too many genes. Finally, the gene sets separately obtained from KEGG, Transfac and GO‐BP were merged to provide 2,697 “Merged” annotations. For details of the above annotation sets see “Supporting Information Methods.”

#### 2.1.5 Choice of tuning parameter

The tuning parameter λ (degree of penalization for LASSO) is chosen by performing 10‐fold Cross Validation. We divide the entire SNP set into training and test sets 10 times for cross‐validation (CV). For each CV replicate, we randomly assign half of the SNPs to the training set and the remaining to the test set. Assignment of SNPs is done in short segments (i.e., preserving stretches of contiguous SNPs) to account for LD structure. We fit our penalized logistic regression model using *glmnet* on the training data and calculate overall power (i.e., expected number of true positives) for the “Simple Weighting” method in the test data for different values of λ. We select the λ for which the maximum power is obtained on an average over 10 sets.

#### 2.1.6 Computation and scalability

We have implemented our algorithm described above into a scalable pipeline within an R package “GKnowMTest.”

**Figure 1:**
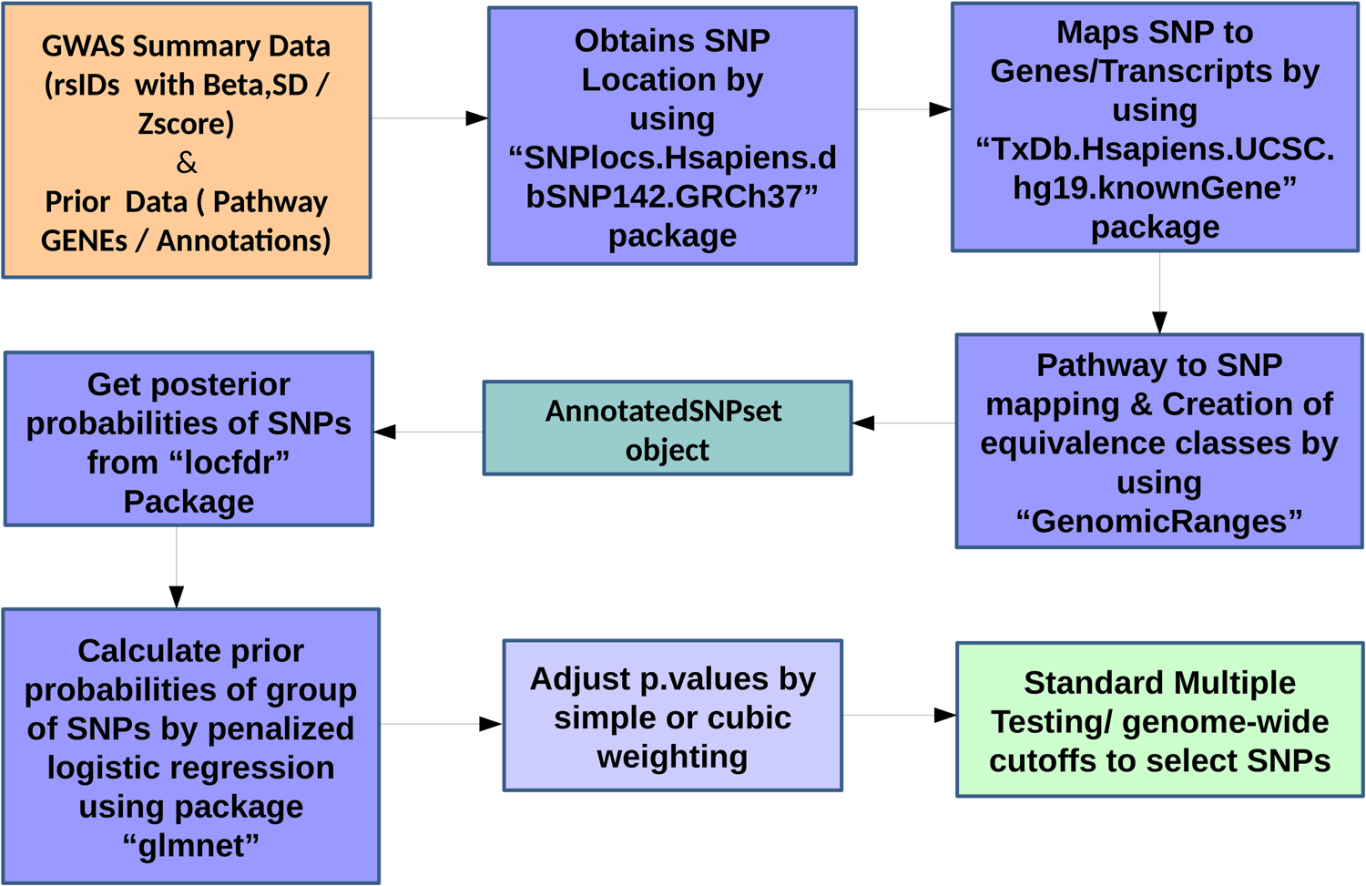
Workflow of R package “GKnowMTest”

The overall flowchart of our package is shown in Figure 1. First, the input SNP list and list of gene‐level annotations is processed bioinformatically to map SNPs onto pathways. Further, SNPs with identical sets of annotations are grouped into equivalence classes. This optional step can provide significant savings in computation time and memory usage. The mappings along with the equivalence information is stored into an “AnnotatedSNPSet” object. The posterior marginals are obtained using the R package *locfdr* (Efron, Turnbull, & Narasimhan, 2015).

The sparse generalized linear model (GLM) in Equation (4) can be efficiently fitted using the R package *glmnet* (Friedman, Hastie, & Tibshirani, 2010) that uses the fast “cyclic co‐ordinate descent” algorithm for fitting. It allows the user to control the type of sparseness penalty and degree of shrinkage through the α and λ parameters. α controls the type of penalty (α=0 for LASSO, α=1 for Ridge and intermediate values 0<α<1 for Elastic NET). For the real data sets which we analyzed, LASSO, Ridge, and Elastic‐Net penalties returned very similar results (results not shown). Hence we used LASSO (i.e., α=1) throughout for our analyses and simulations.

Finally, for Type 1 error allocation with CPW method, unconstrained optimization by Nelder Mead was implemented using the *optim* function in R. To assess the computation times of our Enrichment Estimation (PMLR) to a MLE based approach, we compared the running times with FGWAS software (Pickrell, 2014), although this method is not directly applicable in our context. For the time comparison we used KEGG and a merged combination of KEGG and Transfac annotations.

### 2.2 Whole‐genome simulations

#### 2.2.1 Simulation algorithm

We developed an in‐house algorithm for directly simulating a large number of (independent) causal loci retrospectively for a given number of cases and controls. The algorithm (implemented in R) works by simulating a latent variables for causal loci from multivariate normality with different means in cases and controls, and then thresholding the latent variables to get the genotypes (see “Supporting Information Methods”). In this way, 500 replicates of the *Z*‐scores at the causal SNPs are simulated and then a truly associated region is simulated within a 1 MB neighborhood of the causal SNP, and finally remaining *Z*‐scores are simulated from the null (see “Supporting Information Methods”).

#### 2.2.2 Type 1 error and power

Our simulations were modeled using psoriasis, in the sense that the MAFs and ORs of the causal SNPs and genomic locations were chosen to be close to that of 25 suggestive GWAS loci (i.e., *p* < 1e−05) of psoriasis from the GWAS catalog. The simulations were repeated 500 times to check the Type 1 errors for various levels by treating SNPs outside ±1 MB of the causal SNPs as null SNPs. We used the *Z* scores of the causal SNPs (but not neighboring SNPs) to assess power at $\alpha =10^{-3}$ or $10^{-5}$ or $10^{-7}$.

## 3 RESULTS

### 3.1 Simulation results

To study the Type 1 error and power properties of our method, we simulated 1,500 case and 1,500 control genomes. The results are outlined below.

#### 3.1.1 Type 1 error

First, for the two weighting schemes namely, Simple weights (SPW) and Cubic weights (CPW) as discussed in Secion 2, we checked if the Type 1 errors are maintained at different levels starting from 0.1 to stringent levels 1e−07.

**Figure 2:**
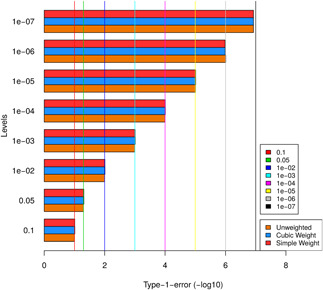
Barplot of Type 1 error for unweighted and weighted *p* values across levels of significance levels. *X* axis shows −log 10 values of Type 1 error achieved and *Y* axis shows the significance level at which each SNP is rejected (target level). The plot shows that the Type 1 error is maintained at different levels of significance

Figure 2 provides the bar plot showing Type 1 errors of unweighted and weighted *p* values for different weighting schemes. It was observed that the Type 1 error for both the SPW and CPW schemes are correctly maintained within target levels. It should be noted that here we considered only global Type 1 error (not SNP specific) averaged over all null SNPs across the genome using 500 whole genome simulations (see also Section 2). At extreme tail (1e−07), the Type 1 error estimates were not very accurate for 500 simulations but the SPW and CPW methods had similar Type 1 error as the “Unweighted” method.

#### 3.1.2 Overall and SNP‐specific power

Overall power of weighted‐analysis (both SPW and CPW) was consistently higher than unweighted analysis across levels of significance (Figure S1). The power of the two alternative weighting schemes is similar, although cubic weights (CPW) performed slightly better than simple weights (SPW) at lower levels. In terms of power for specific SNPs (Figure S2), we found that while some SNPs gain power, some are essentially unaffected while a few of them lose power. It is evident from the figure that the gain in power is much more both in terms of magnitude and number of causal SNPs compared to the loss of power. Isolated true SNPs (not connected to other true SNPs through supplied annotations) are expected to lose power. Thus supplying better annotations in pathway‐guided search can help in discovering additional loci missed by primary unbiased GWAS (see Section 4).

#### 3.1.3 Connectedness and power

**Figure 3:**
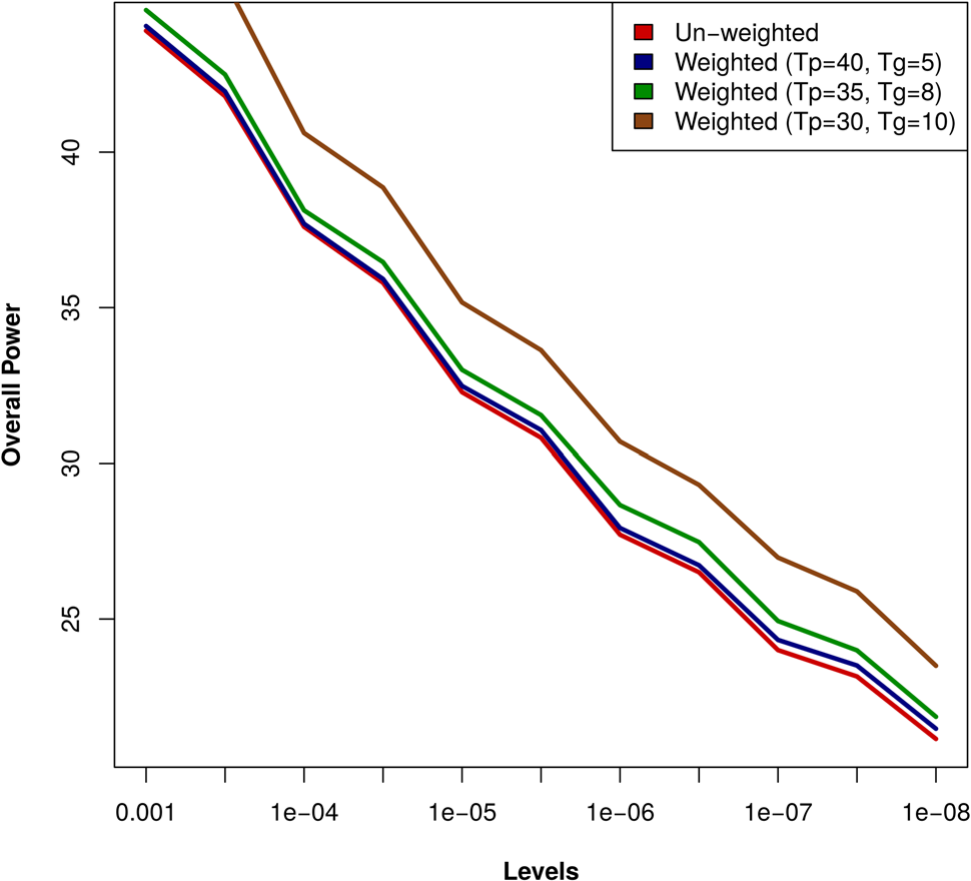
Change of overall power curve with connectivity of genes using synthetic pathway lists. $T_{p}$ denotes number of “true pathways” (selected to be enriched) and $T_{g}$ denotes number of true genes allocated to each “true pathway.” *X* axis shows levels of significance and *Y* axis shows overall power. Black, red, green, and blue lines show overall power across significance levels, respectively, for unweighted analysis, first synthetic pathway list ($T_{p}=40$, $T_{g}=5$, i.e., low connectivity), second pathway list ($T_{p}=35$, $T_{g}=8$, i.e., moderate connectivity) and third pathway list ($T_{p}=30$, $T_{g}=10$, i.e., high connectivity among true genes)

It is expected that when a causal gene is “more related” to other causal loci through pathways, it should have a higher power of being detected. The prioritization method works better when more causal SNPs cluster into a smaller number of pathways. To illustrate this, we generated three pathway lists with increasing degree of connectivity among “truly associated” genes (see “Supporting Information Methods” for details. Figure 3 shows power curves for these three lists with $T_{p}$ denoting “number of true pathways” (selected for enrichment) and $T_{g}$ denoting the “number of true genes” allocated to each such pathway. We found that the overall power of the study is considerably higher for the “moderately connected” list and highest for the “highly connected” pathway list. The increase of power with connectivity is maintained across all significance levels. Further, we did simulations with increasing connectivity among a randomly selected set of “null genes” (instead of “true genes” used for power simulations), and confirmed that there is no inflation of global Type 1 error (Figure S3). These results show that when the causal loci are more connected to each other in pathways, they contribute to the enrichment of the pathway and thus increase the overall power of detecting causal SNPs from weighted analysis.

We used real GWAS data sets to show the efficiency of our method in identification of novel loci that were otherwise lost due to the stringent genome‐wide threshold. We used summary level data of four different diseases namely psoriasis, SLE, coronary artery disease (CAD) and type 2 diabetes. The type 2 diabetes data results are explained below. Results for the other diseases are described in “Supporting Information Results” section.

#### 3.1.4 Shrinkage and power

It is crucial to estimate the degree of penalization from the data, because if penalty is over‐specified or under‐specified it can lead to loss of power. To verify this, we estimated power of the causal SNPs for a sequence of λ values (the default sequence of λ values from *glmnet*) for the LASSO penalty.

**Figure 4:**
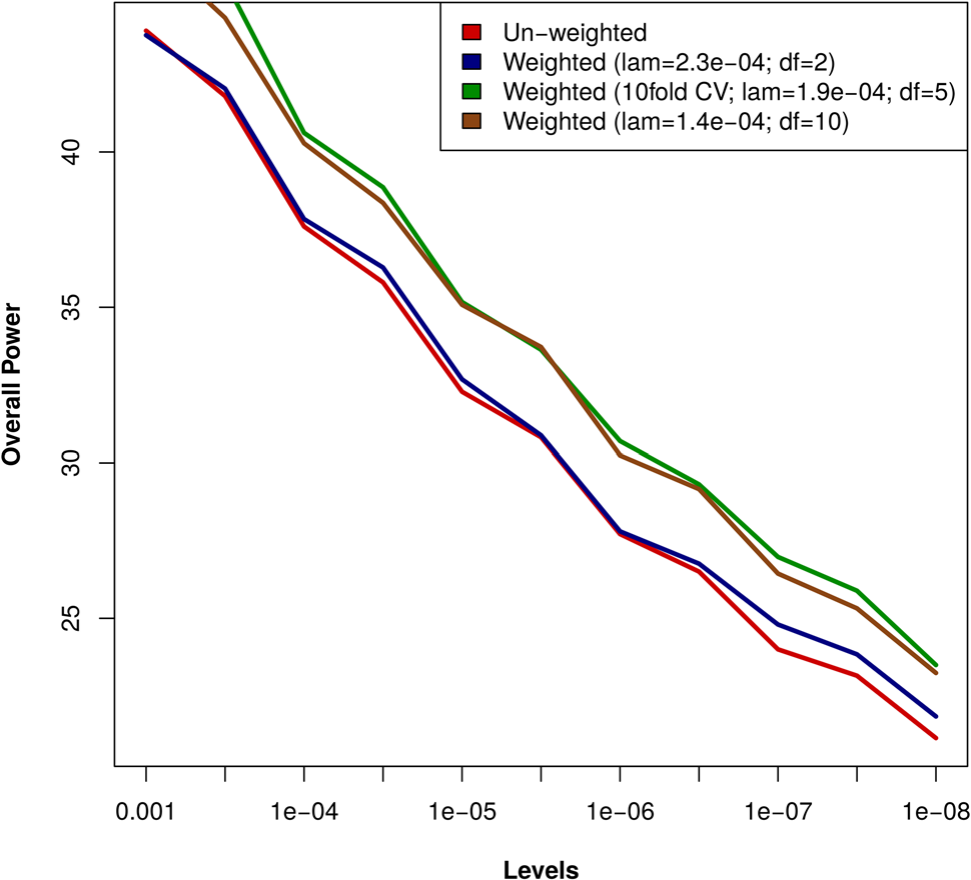
Change of overall power curve with Lasso penalty. X axis shows levels of significance and *Y* axis shows overall power. Black and green solid lines show power for unweighted and weighted analysis (*df* = 5; Lasso with 10‐fold CV). The red line shows power when weights were derived with λ corresponding to fewer degrees of freedom (*df* = 2) than the optimum λ and the blue line shows power when weighted with λ corresponding to higher degrees of freedom (*df* = 10) than the optimum λ thus leading to over‐fitting

Figure 4 shows that the overall power of detection is close to that of “unweighted analysis” for large λ values (i.e., low degrees of freedom) and increases gradually with decreasing λ (i.e., higher degrees of freedom). This is because when λ is too large, the weights shrink towards zero, and the PMLR method becomes essentially similar to “unweighted analysis” which has lower power. However, for very small λ values overfitting can result in loss of power. The figure shows that weighted analysis with cross‐validation avoids over‐fitting and has the highest power overall across levels.

### 3.2 Analysis of real GWAS data sets

#### 3.2.1 Time comparison with MLE

It is of interest to look at the computational speed‐up achieved using PMLR instead of full‐likelihood maximization generally used in other tools. We used FGWAS (Pickrell, 2014) and our method to run the real data of psoriasis. The running time for FGWAS for 229 KEGG pathways was 3 h 40 min while our method took 10 min 37 s to run on the same data. Next we merged KEGG and TRANSFAC annotations (376 annotations in total) and compared the running times. FGWAS took 4 h 43 min while our method 10 min 41 s for the same set of SNPs and annotations. For the above comparisons we used a linux computer with i7‐5500U CPU and quad processor of 2.40 GHz and 16 GB DDR3 RAM running Ubuntu 16.04 LTS. Thus our PMLR approach works much faster than full‐likelihood maximization implemented in a popular existing SNP‐prioritization method (FGWAS).

#### 3.2.2 *p* value weights and shrinkage

Using the summary results obtained from the analysis of psoriasis data (Tryka et al., 2013) downloaded from dbGAP (described in “Supporting Information Methods”), we studied the behavior of *p* value weights generated by our method with change of penalty (λ). For this, we considered KEGG pathways (Kanehisa & Goto, 2000; see Section 2) as annotations. The input *p* values were transformed to *Z*‐scores as $Z_{j}=\Phi^{-}1\left[1-P_{j}\right]$. The PMLR method was applied using the R packages *locfdr* to obtain mPPA (Marginal Posterior Probability of Association) values and *glmnet* for penalized logistic regression using LASSO penalty (i.e., α=1). A decreasing sequence of λ values was used to derive priors and hence *p* value weights (using the Cubic Weighting, i.e., CPW method).

**Figure 5:**
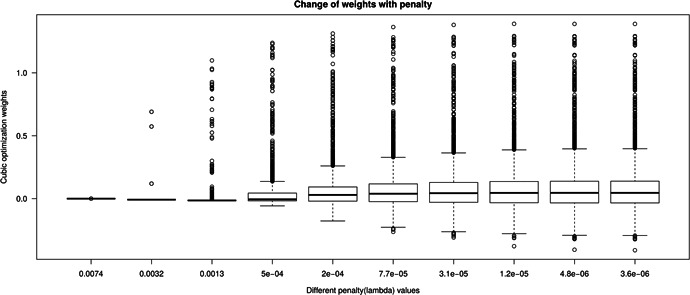
Box plots of weights with change in LASSO penalties. *X* axis gives the LASSO penalty λ and *Y* axis gives the *p* value weights (based on cubic optimization). The plot shows increase in the spread (variability) of *p* value weights with decrease in penalty. For the highest penalty all weights are shrunk to 1 (unweighted analysis)

Figure 5 shows change in the spread of *p* value weights (in the log10 scale) with various λ values for LASSO penalty. As expected, the weights have increasing variability around 1 as the degree of shrinkage (λ) decreases. For the largest λ value, the coefficients are all shrunk to zero and hence the weights are all 1 (i.e., reduces to unweighted analysis).

#### 3.2.3 Comparison among different annotation sets

We conducted reweighted analysis of the summary results from psoriasis GWAS with KEGG, Transfac, GO (BP) and MERGED annotations separately and inspected 18 known psoriasis associated SNPs (i.e., *p *< 5e−08) from the GWAS catalog (MacArthur et al., 2017) that were genotyped in our data. The unweighted *p* values and weighted *p* values based on all four annotations are shown in Table 1, sorted by unweighted *p* value. As seen from this table reweighted *p* values are smaller than unweighted *p* values in many cases (shown by bold) except toward the bottom of the table, containing SNPs for which there is no association signal in this data. Further the SNP rs20541 mapping to IL13 gene become genome‐wide significant using the MERGED annotation. The results for “MERGED” seem to be dominated by “GO.BP” which has relatively much larger number of annotations but there were exceptions such as rs492602 SNP in the FUT2 gene. Overall, the MERGED annotation set was the most powerful giving close to smallest *p* values in most cases. Thus, in situations where there are multiple annotations with different kinds of information, creating a pooled annotation set (such as “MERGED”) may be effective. We used the MERGED annotation set for all the reweighted GWAS analyses in this article. It should be noted that “MERGED” is just an example of a generic annotation set, such annotations sets can be defined in many different ways. However, a suitable set of annotations to be used for a specific reweighted GWAS analysis should be predetermined by the investigator rather than using a trial‐and‐error approach (see Section 4).

**Table 1:** Results of reweighted analysis of psoriasis GWAS data for 18 known psoriasis associated SNPs using four different annotation sets

SNPs	Genes	Unweighted	KEGG	TRANSFAC	GO.BP	MERGED
rs12191877	HLA‐C	6.47E−22	4.91E−23	6.65E−22	**1.89E**−**23**	2.06E−23
rs2082412	IL12B	1.97E−08	5.18E−09	5.16E−09	2.20E−09	**2.04E**−**09**
rs20541	IL13	5.98E−07	5.00E−08	8.66E−08	4.63E−08	**3.88E**−**08**
rs17728338	TNIP1	9.25E−05	1.10E−04	**9.12E**−**05**	1.07E−04	1.16E−04
rs240993	TRAF3IP2	2.64E−04	2.83E−04	1.99E−04	1.88E−04	**1.82E**−**04**
rs2201841	IL23R	3.72E−04	3.70E−04	2.78E−04	**7.63E**−**05**	8.08E−05
rs2066807	IL23A	4.71E−04	3.47E−04	3.01E−04	**1.06E**−**04**	1.49E−04
rs610604	TNFAIP3	5.06E−04	4.96E−04	5.09E−04	2.85E−04	**2.81E**−**04**
rs11795343	DDX58	2.89E−03	2.58E−03	3.21E−03	**1.55E**−**03**	1.75E−03
rs11053802	KLRK1	1.50E−02	1.83E−02	**1.29E**−**02**	1.47E−02	1.60E−02
rs492602	FUT2	3.15E−02	1.69E−02	1.45E−02	1.85E−02	**6.26E**−**03**
rs1990760	IFIH1	9.39E−02	9.96E−02	9.65E−02	**3.59E**−**02**	5.17E−02
rs4795067	NOS2	**1.11E**−**01**	8.89E−02	8.25E−02	1.36E−01	1.41E−01
rs4085613	LCE3D	1.85E−01	2.04E−01	1.90E−01	**1.50E**−**01**	1.64E−01
rs2944542	ZNF365	**2.26E**−**01**	2.50E−01	2.33E−01	2.64E−01	2.64E−01
rs9513593	UBAC2	**2.86E**−**01**	3.15E−01	2.93E−01	4.75E−01	4.70E−01
rs10789285	LRRC7	**4.66E**−**01**	5.15E−01	4.79E−01	5.43E−01	5.43E−01
rs27524	ERAP1	6.27E−01	6.92E−01	**3.47E**−**01**	4.62E−01	4.04E−01

*Note*: Bold font indicates lowest *p* value for each row.

#### 3.2.4 DIAGRAM consortium T2DM data

We downloaded summary data on type 2 diabetes from the DIAGRAM (Diabetes) consortium website. These data were based on the stage 1 meta‐analysis of 8,130 T2DM cases and 38,987 T2DM controls of from European population (Voight et al., 2010). Unweighted analysis showed 12 GWS loci. Pathway guided GWAS with the MERGED annotation set selected 166 annotations (i.e., pathways) upon cross‐validation and gave six “crossover” loci (i.e., loci that were not GWS in the unweighted GWAS analysis, but became significant after weighting).

**Figure 6:**
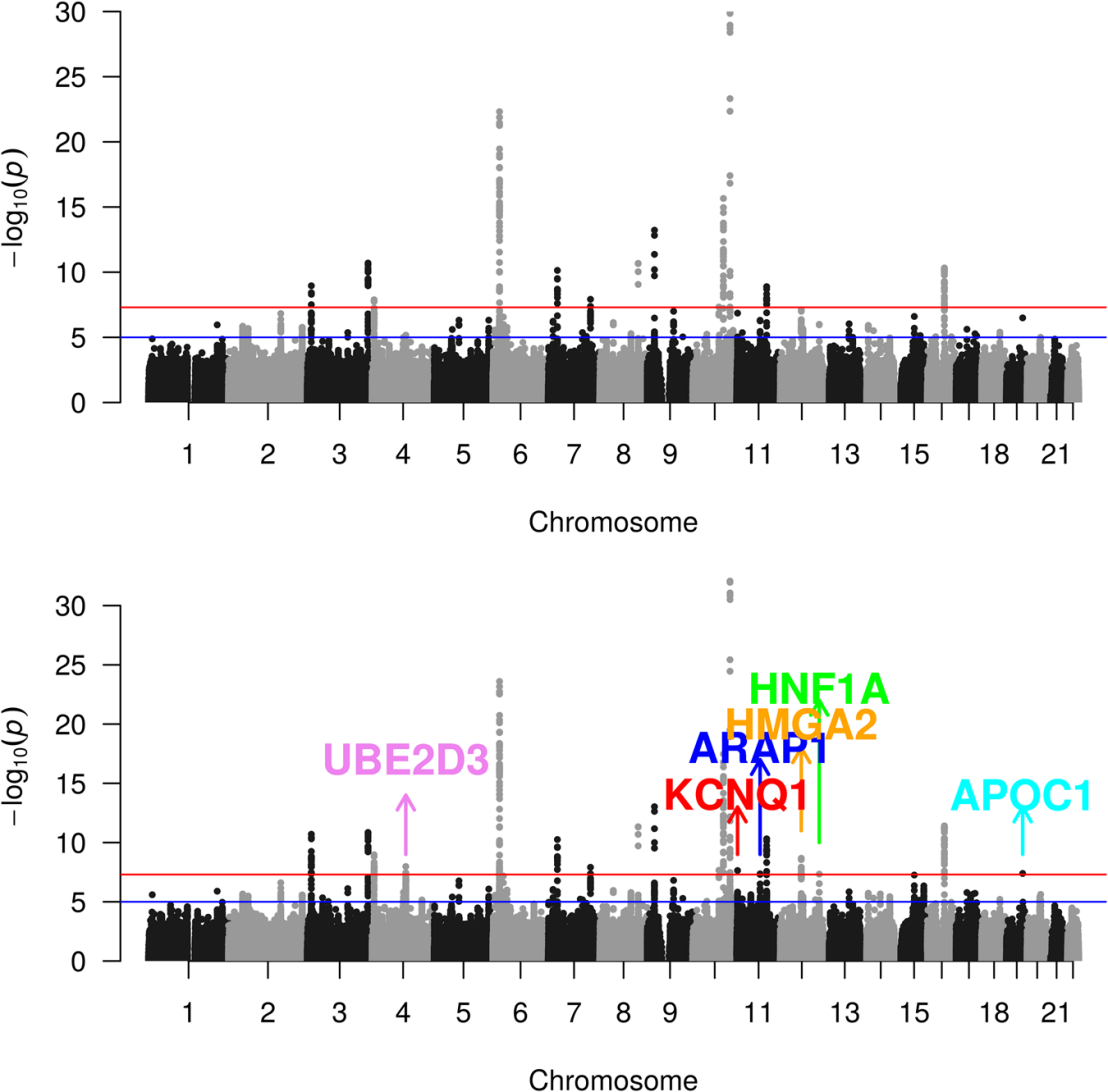
Manhattan plots of type 2 diabetes before and after weighted analysis. Upper and lower panels denote Manhattans of unweighted *p* values and *p* values weighted by MERGED annotation set (with cubic weighting). Two crossover loci (i.e., region newly detected by weighted analysis) are shown with names of genes mapping to those regions

The Manhattan plots before and after weighting are showed in Figure 6. There was one crossover locus near UBE2D3 gene on 4q24 (lead SNP rs223340). This SNP has been reported as eQTL for a lncRNA gene LRRC37A15P (Bhalala, Nath, Inouye, & Sibley, 2018). It is also close to a missense variant in CISD2 gene which causes Wolfram Syndrome 2 (Rouzier et al., 2017). Three distinct loci were identified on chromosome 11. The locus on 11p15 was near KCNQ1 gene (lead SNP rs231362). This SNP has been reported in the same article by Voight et al. (2010) from the Stage‐2 GWAS comprising 34,412 cases and 59,925 controls and recently by Zhao et al. (2017). Another locus on 11q13 was near the ARAP1 gene (lead SNP rs11603334). The SNP has been reported previously as GWS for “proinsulin levels” (Strawbridge et al., 2011) and “lycated hemoglobin” (Wheeler et al., 2017). A locus on 12q14 (lead SNP rs2612069) was near the HMGA2 gene. SNPs in this region (e.g., rs343092 within 300 kB) have been reported previously for T2DM (Ng et al., 2014). Another chromosome 12 SNP is rs11020107 on 12q24. This has been previously found (Morris et al., 2012). The last SNP detected was on chromosome 19 rs4420638 on 19q13, near APOC1 gene. This SNP has previously been associated with type 2 diabetes by Zhao et al. (2017).

We used the MERGED annotation set to perform re‐weighted GWAS analysis on other disease data sets (described in Figures S4, S5, and S6). We found one crossover locus of IL13 for psoriasis, two crossover loci BLK and ITGAM/ITGAX genes for SLE and four crossover loci near TFPI, CYP2A1, PECAM1, and CXCL12 for CAD data set. Unweighted and weighted *p* values for these SNPs along with the association results from some independent studies are summarized in Table S1.

## 4 DISCUSSION

In this article, we introduced a new efficient analytic pipeline to enable pathway‐guided search in a GWAS. Using this pipeline we illustrated that substantial power improvement can be achieved in GWAS by using knowledge of biologically meaningful categorization of genes, for example, pathways, ontology terms and so forth. The power gain is highlighted by the “novel loci” (i.e., crossover loci) detected in the pathway‐guided reanalyses of real GWAS data sets. Using, whole‐genome simulations, the “pathway‐guided GWAS” is shown to have correct global false‐positive rate, and improved power for well connected SNPs. As expected, there is loss of power for isolated SNPs, but we found such power loss to be less common and also much less in magnitude. Moreover, with better prior annotation strategies and as annotation databases become more comprehensive and accurate, most “isolated” true SNPs are likely to be connected with other causal genes, further reducing this concern. Isolated SNPs involving novel biological mechanisms (without known connections in annotation databases) would have higher power to be detected by primary (unbiased) GWAS analysis.

Our pipeline consists of a new method called PMLR for adaptively estimating “enrichments” (i.e., prior probabilities) followed by optimal *p* value weighting to control FWER. The primary advantage of PMLR over existing methods for enrichment estimation in GWAS is that it involves two simple steps, local FDR calculation followed by a (penalized) logistic regression. Thus it can be implemented using standard GLM and penalized GLM software in any statistical package. Also, compared to some existing prioritization algorithms requiring high‐dimensional MLE or posteriors, it is faster, scalable to large number of annotations and less likely to encounter numerical problems such as nonconvergence, making it suitable for routine use.

A limitation of our method, which is not specific to the method proposed here, is that the local FDR approach ignores the association (i.e., LD) structure of the SNPs in deriving the marginal PPAs. To our knowledge, all other existing prior‐incorporation methods assume independence across SNPs or SNP blocks. In the future, we plan to overcome this limitation by allowing for correlations among SNPs in the mPPA estimation step in a computationally efficient manner. A key benefit of our modular approach is that our pipeline can be easily adapted to be used in conjunction with other alternative methods for mPPA estimation and/or Type 1 error allocation methods in future.

We have incorporated gene‐level prior knowledge only. Our method is not comparable to the existing prioritization methods. To our knowledge, no existing method incorporates gene‐level priors (e.g., pathways) using a conventional *p* value threshold approach. Second, we have developed a statistically powerful method simply from pathway knowledge (gene‐level knowledge), without the use of more detailed information such as SNP‐level annotations. Existing SNP prioritization methods make assumptions such as “one true SNP per region” to identify causal variants, so they are not applicable (without modifications) in our context, that is, discovery of “associated SNPs” using gene‐level priors. In the future, a careful study of SNP‐level annotations and pathways would be required to understand the best way of combining these two sources of information in a scalable manner.

The list of pathways supplied to our method should generally not be restricted to “candidate pathways” thought to be important for the disease under study. An unbiased list of pathways or “gene sets” (possibly a large number) representing meaningful biological categories may be supplied. Depending on a disease context, important categories of genes (e.g., cancer related genes in a cancer GWAS) may be added to the pool of annotations to potentially improve power. However, restricting genes (e.g., removing noncancer genes from annotations) can reduce power. Also, it should be recognized that reweighted analysis of a GWAS data set is as likely as the original GWAS to yield a false‐positive finding. The chance of making a false‐positive discovery is further enhanced if an investigator explores multiple annotation databases until a discovery is made. To guard against such false‐positives, we recommend that an investigator should predetermine the annotations to be used and also follow stringent criteria for replication and validation to confirm a novel finding.

In conclusion, we have provided a framework and have developed a method that can enable routine use of pathway and other gene‐level annotations for prioritization in GWAS. Pathway‐guided GWAS can be effectively used in practice for secondary reweighted GWA scan to make additional discoveries beyond those revealed by the primary unbiased GWAS analysis. Our method allows flexibility to investigators to either use standard pathway or ontology databases or define their own gene sets. Being modular in nature, it allows for future extensions to more genomic data types and bioinformatic knowledge from multiple sources.

## Supporting information

Supplementary Information

## Data Availability

Data sharing is not applicable to this article as no new data were created or analyzed in this study.
