# Supplementary material for: A framework for pathway knowledge driven prioritization in genome‐wide association studies
Source: Genet Epidemiol. 2020 Aug 10;44(8):841–53. doi: 10.1002/gepi.22345 (PMC7116354; doi:10.1002/gepi.22345)
Supplement: Supplementary file 1 — Supplementary Information [file GEPI-44-841-s001.pdf]

MANUSCRIPT SUBMISSION

SUPPLEMENTARY MATERIAL

A Framework for Pathway Knowledge Driven Prioritization in Genome-wide Association Studies

Contents

S1 Supplementary Methods **2**
S1.1 Derivation of Two-step Prior Estimation (PMLR) 2
S1.2 Type-1 Error Allocation 2
S1.3 Retrospective Simulation of Causal SNPs 3
S1.4 Whole-genome Simulation Scheme 3
S1.4.1 Pathway Allocation 3
S1.5 Analysis of Psoriasis GWAS Data 4
S1.6 Deriving KEGG Pathways 4
S1.7 Deriving Transfac TF Target-Gene Sets 4
S1.8 Deriving Gene-Ontology (Biological Processes) Annotations 4
S1.9 Deriving a Merged Annotation Set 4

S2 Supplementary Results **6**
S2.1 Simulation Results 6
S2.1.1 Overall Power 6
S2.1.2 SNP specific power 6
S2.1.3 Connectedness among Null Genes 6
S2.2 Re-weighted Analyses of GWAS Summary Results 6
S2.2.1 Re-weighted GWAS of Psoriasis 8
S2.2.2 GWAS of SLE (Systemic Lupus Erythematosus) 9
S2.2.3 GWAS of Coronary Artery Disease (CAD): 10

S3 Data Sources **15**

S1 Supplementary Methods

S1.1 Derivation of Two-step Prior Estimation (PMLR)

Here we give intuitive justification of the PMLR method only for the case of $K < M$ and without any regularization. For brevity denote $\theta = (\kappa, \beta)$ and $X = [1\ V]$. Assuming -s to be known, the (complete data) log-likelihood for the logistic model is

$$l(\theta) = \sum_{j=1}^{M} \delta_j * \log \frac{1}{1 + exp(-\theta' X_i)} + (1 - \delta_j) * \log \frac{1}{1 + exp(\theta' X_i)}$$

The expected log-likelihood is

$$Q(\theta|\theta^t) = \sum_{j=1}^{M} Pr(\delta_j = 1|Z_j, \theta^t) * \log \frac{1}{1 + exp(-\theta' X_i)} + [1 - Pr(\delta_j = 1|Z_j, \theta^t)] * \log \frac{1}{1 + exp(\theta' X_i)}$$

Thus the M-step (without penalty) is simply a logistic regression. In the first step, assuming $\kappa^0 = log(\hat{\pi_1}/\hat{\pi_0})$ and β-s to be 0, i.e. $\theta^0 = (\kappa^0, 0, \dot{,} 0)$, the $Pr(\delta_j = 1|Z_j, \theta^0)$ reduces to $Pr(\delta_j = 1|Z_j)$ and hence 1 st M-step reduces to

$$= \sum_{i=1}^{C} n_i * \left\{ \psi_i * \log \left(\frac{1}{1 + exp(-\theta' X_i)} \right) + (1 - \psi_i) * \log \left(\frac{1}{1 + exp(\theta' X_i)} \right) \right\}$$

The above expression justifies the reduction of Equation (1) involving SNPs to Equation (3) involving equivalence classes, which gives considerable computational saving (both storage and speed) when $C << M$. Both the glm and glmnet functions allow $\bar{\psi_i}$ as response with n_i as weights. Clearly the method can be interpreted as 1-step EM starting with a reasonable initial estimate of the posteriors under $\theta = \theta_0$ (that is itself a solution of an MLE). Further this posterior becomes more accurate with sample size as the information from the data Z dominates that of the prior for large sample sizes. Due to this consistency of the posterior [i.e. $Pr(\delta|Z) \Rightarrow \delta$], $\hat{\kappa} = (\hat{\kappa}, \hat{\beta})$] the solution of the logistic regression in Equation (3) converges to the solution of the logistic regression of δ on X, which is the MLE and hence a consistent and asymptotically efficient estimator of (γ, η) in Model (1).

S1.2 Type-1 Error Allocation

We consider the maximizing the expected number of true positives among all the tests constraining the expected number of false positives (under the global null) to the desired FWER level ($\alpha = 0.05$). Let $w_i\alpha$ denote the total type-1 error to be allocated for SNPs in the ith equivalence class. These weights w_i should add to 1 for the FWER to be maintained at α ((Roeder, Devlin, & Wasserman, 2007)). Then the type-1 error spent on each SNP in that class (assuming Bonferroni correction) becomes $\frac{w_i \alpha}{n_i}$. Define $w'_i = w_i * \frac{M}{n_i}$ are SNP-level weights within each equivalence class.

Thus, $p'_j = min(1, p_j/w'_i)$ can be viewed as a re-weighted p-value. The expected number of true positives is:

$$E(TP) = h(w'_1, w'_2, \ldots, w'_C) = \sum_{i=1}^{C} n_i\ \hat{\pi}_i\ \overline{F_1} \left\{ \overline{\Phi}^{-1} \left[\frac{\alpha w'_i}{M} \right] \right\} \quad (1)$$

Using a Lagrange multiplier for the type-1 error constraint, we need to maximize

$$g(w', \psi) = h(w') + \gamma.\{\overline{w'} - 1\},$$

where $\overline{F_1}$ and $\overline{\Phi}$ denote the null and alternative upper tail CDFs of the Z-scores.

S1.3 Retrospective Simulation of Causal SNPs

We assume independent latent Gaussian variables $G_{j1}, G_{j2}, j = 1, \ldots, m$ corresponding to minor allele indicators for 2 alleles each from 'm' causal SNPs, and these are $iid\ N(\mu_j, \sigma_j^2)$ under the additive model. Assume that an individual's 1_{st} allele at SNP j is the minor allele if $G_{j1} > 0$ and absent otherwise. Similarly 2_{nd} allele is minor if $G_{j2} > 0$. The parameters $\mu_j and \sigma_j$ are chosen to satisfy the required MAF condition and the equation $E(G_{j1}|G_{j1} > 0) - E(G_{j1}|G_{j1} < 0) = 1)$. The model assumed is $logit[Pr(D_i = 1|\omega_i)] = a + \omega_i$ where $\omega_i = \sum_{j=1}^{m} b_j * (G_{j1i} + G_{j2i})$. Here b_j is the desired per-allele log-odds ratio of SNP j for the usual logistic regression on additively coded genotypes. The intercept a is determined by solving for the prevalence of the disease. Note that the population distribution of the latent liability ω_i is normal. First the liability ω is retrospectively simulated within cases and controls with its posterior distribution determined by Bayes' Theorem. Next the latent Gaussian variables G_{j1} etc are simulated from a (singular) multivariate normal conditional on ω. Finally, the simulated Gaussians are thresholded at 0 to give the simulated alleles and genotypes.

S1.4 Whole-genome Simulation Scheme

After the causal genotypes are simulated, a chi-square test of association is done to obtain two-tailed p-values which are inverted to give Z-scores at the causal SNPs. Thus, expected (mean) Z-score values at the causal loci are derived. Next, the mean of the SNPs in LD with the causal SNPs are calculated using a linear decay factor based on the distance of the neighbouring SNPs from the causal SNPs. We considered a steep linear decay in LD upto 10kb on both sides of the causal SNP ($r^2 = 0.1$) and a further gradual decay to ($r^2 = 0$) stretched till 30 kb on both sides. The remaining SNPs in the genome are considered to be from the null distribution (standard normal). In this way summary Z-scores are directly simulated for, 500 replicates for the entire genome.

S1.4.1 Pathway Allocation

For the type-1 error and power simulations we used synthetic pathways where genes were grouped in a manner so that the degree of connectivity between the 'truly associated genes' could be controlled. Suppose, T_p denotes 'number of true pathways' (selected for enrichment) and T_g denotes the 'number of true genes' allocated to each such pathway. We calculated overall power curve across significance levels for 1) unweighted analysis and weighted analyses with three different pathway lists, i.e. 2) Low connectivity ($T_p = 30, T_g = 10$) 3) Moderate connectivity ($T_p = 30, T_g = 10$) and 4) High connectivity ($T_p = 30, T_g = 10$). Thus, in the first list, the causal genes are sparsely distributed, while the second and third gene lists had more overlaps between 'true genes' within pathways. All the type 1 error plots were generated using data from the third gene list i.e. with $T_p = 30$ and $T_g = 10$. The total number of causal SNPs, based on which power is calculated is the same in each case. The total number of causal SNPs, based on which power is calculated is the same in each case.

S1.5 Analysis of Psoriasis GWAS Data

We downloaded GWAS data on Psoriasis consisting of 1642 European ancestry subjects (950 psoriasis cases and 692 controls) from the Collaborative Association Study of Psoriasis via the database of genotypes and phenotypes (dbGAP) (Tryka et al., 2013). The original study comprised 1409 cases and 1436 controls. The subset we analysed were those in the 'General Research Use' consent group available from dbGAP (dbGaP Study Accession: phs000019.v1.p1). We used Eigenstrat (Price et al., 2006) to derive principal components and selected top 4 PC-s to represent ancestry. For GWAS analysis, R was used for logistic regression with additively coded SNP genotypes adjusted for principal components.

S1.6 Deriving KEGG Pathways

We used R/Bioconductor package *org.Hs.eg.db* (Carlson, 2017a) to generate 229 KEGG pathways (Kanehisa & Goto, 2000). The mapping between KEGG pathway identifiers and Entrez gene identifiers was obtained from the '*org.Hs.egPATH*' object in this package. Entrez gene identifiers were replaced by entrez gene symbols by using the '*org.Hs.egGENENAME*' object. Names of KEGG pathways was obtained from the KEGG.db package (Carlson, 2016).

S1.7 Deriving Transfac TF Target-Gene Sets

We obtained data on transcription factors and their validated targets from Transfac (Matys et al., 2006). We processed the data from the flat files and used CRAN Package igraph (Csardi & Nepusz, 2006) to create an igraph object. Vertices of this graph were gene symbols of TF encoding genes and their target genes. An edge in the graph says, $A - - > B$ implies that a TF (with encoding gene 'A') targets gene 'B'. To create Transfac annotations, we considered each TF-encoding gene and all genes in its order 1 neighbourhood as a single pathway. This graph had 3228 genes as vertices among which 562 genes are transcription factor encoding genes. We found total 142 different Transfac annotations that had 10 or more genes.

S1.8 Deriving Gene-Ontology (Biological Processes) Annotations

We created GO annotations from GOTERMs of Gene Ontology (Ashburner et al., 2000) Biological Process (GO-BP) domain by using two Bioconductor packages *GO.db* (Carlson, 2017a) and *org.Hs.eg.db* (Carlson, 2017b). We used an algorithm to prune the GO-BP tree to create the annotations. The algorithms works by traversing the relatively large (having >50 genes) offspring nodes downwards. The smaller offspring-nodes are not traversed, but merged with their parent node to create an 'annotation term'. We started with 29586 BP Terms and after pruning we were left with 2570 nodes. Finally, excluding nodes with fewer than 10 or more than 500 genes, we obtained 2326 GO-BP annotations.

S1.9 Deriving a Merged Annotation Set

The gene sets separately obtained from KEGG, Transfac and GO-BP were merged together to give 2697 annotaions. The equivalence classes of each annotation set were used to define a new series of equivalence classes. The advantage of this approach is that the total storage simply adds up (3 separate design matrices),

with an extra matrix for mapping between the merged-equivalence class labels to the original equivalence class labels. For example, if the lth new equivalence class in the merged set denotes SNPs that belong to i^{th} equivalence class in KEGG, j^{th} equivalence class in Transfac and k^{th} equivalence class in GO-BP, then the only additional information stored is $l \longrightarrow (i, j, k)$.

S2 Supplementary Results

S2.1 Simulation Results

S2.1.1 Overall Power

After confirming that the type-1 error is maintained, we looked into the overall power of the weighting schemes using KEGG pathways as annotations. The power was checked for different levels, from 0.1 to stringent GWAS significance level 5e-08. The 'overall power' was defined as 'average number of SNPs crossing the level of significance' or equivalently the 'average proportion of simulation replicates in which a causal SNP crossed the level of significance'. Overall power was calculated at different levels for both un-weighted and weighted p-values (SPW and CPW). **Figure** S1 shows the overall power for SPW and CPW at different levels of significance. Power of weighted analysis was consistently higher than unweighted analysis. The power of the two alternative weighting schemes is similar, although cubic weights (CPW) performed slightly better than simple weights (SPW) at lower levels. This is possibly because power of CPW is optimized at a genome-wide Bonferroni threshold.

S2.1.2 SNP specific power

We also studied the causal SNP specific power from the simulation results. **Figure** S2 shows the bar plot of SNP-wise power for 1) unweighted analysis and 2) weighted analysis (CPW). The powers are shown a genome-wide level of 1e-05 (results for 10^{-3} levels 10^{-7} and were qualitatively similar). In this plot, the causal SNPs are sorted based on decreasing value of difference between power of unweighted p-values and weighted p-values. The plot shows that while some SNPs gain power, most are essentially unaffected while few of them lose power. It is evident from the figure that the gain in power is much more both in terms of magnitude and number of causal SNPs compared to the loss of power. This pattern is to be expected in most realistic scenarios as causal SNPs of a disease would generally map to similar annotations.

S2.1.3 Connectedness among Null Genes

We have shown that if the annotations provided have high connectivity among the true genes, the power of the study increased. We have alongside checked that even if the true genes are highly connected, the type I error remains maintained with an increase in power. We have also checked for the type I error in case the null genes are highly connected in the annotations used S3. As we expected, even with the increase in connectivity among the null genes, the type I error is conserved.

S2.2 Re-weighted Analyses of GWAS Summary Results

We conducted re-weighted analyses of summary results (i.e. p-values) from four GWA studies using our approach and in each case identified one of more loci (i.e. regions) that were missed by unweighted analysis. For all these datasets, we analysed the Z-scores (derived from p-values as $Z_j = \phi^{-1}[1 - P_j]$) using the 'Merged' annotations. We used PMLR with Lasso penalty (10-fold CV) followed by cubic weighting (CPW). Most of these putatively 'novel' variants were identified from other studies independently and/or by the same study by using a larger set of samples. Unweighted and weighted p-values for these SNPs along with the

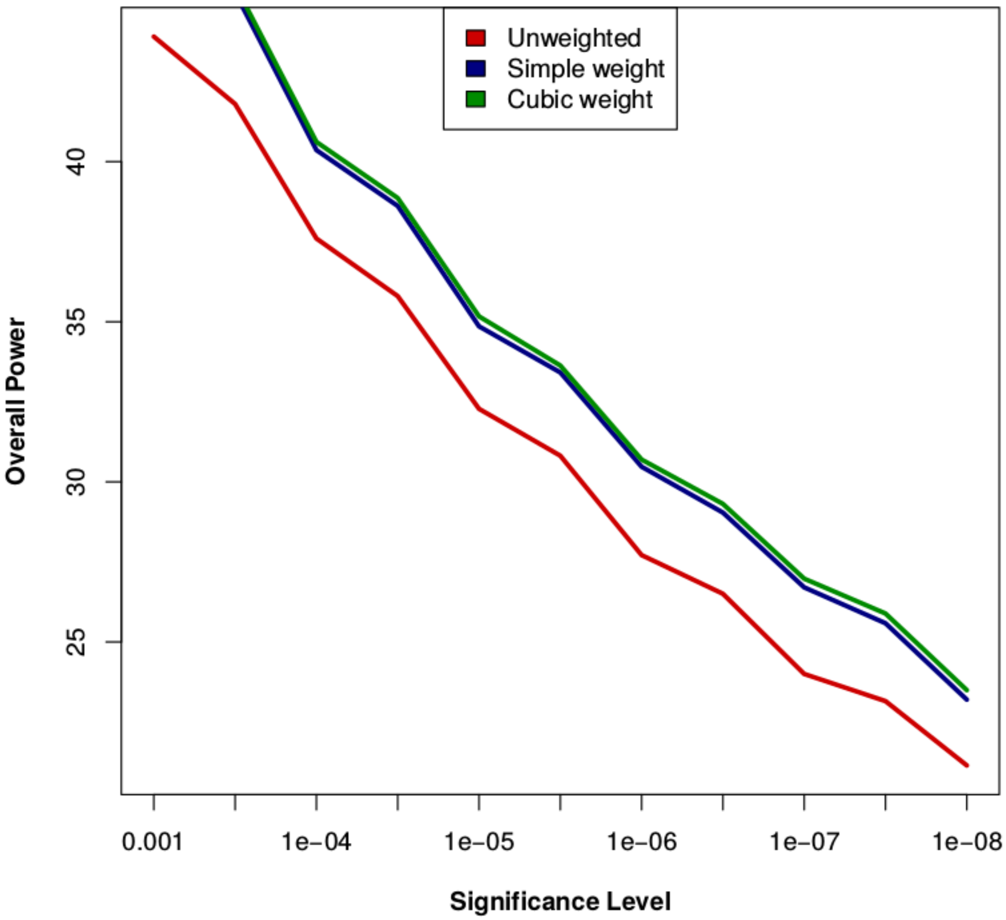

Figure S1: Overall power curve for unweighted and weighted p-values for different weighting schemes at different levels of significance. X-axis shows levels of significance and Y-axis shows overall power (i.e. average power of 25 causal SNPs) based on 500 simulations. The red, blue and green lines show respectively the power for unweighted analysis, simple weighting (SPW) and cubic weighting (CPW).

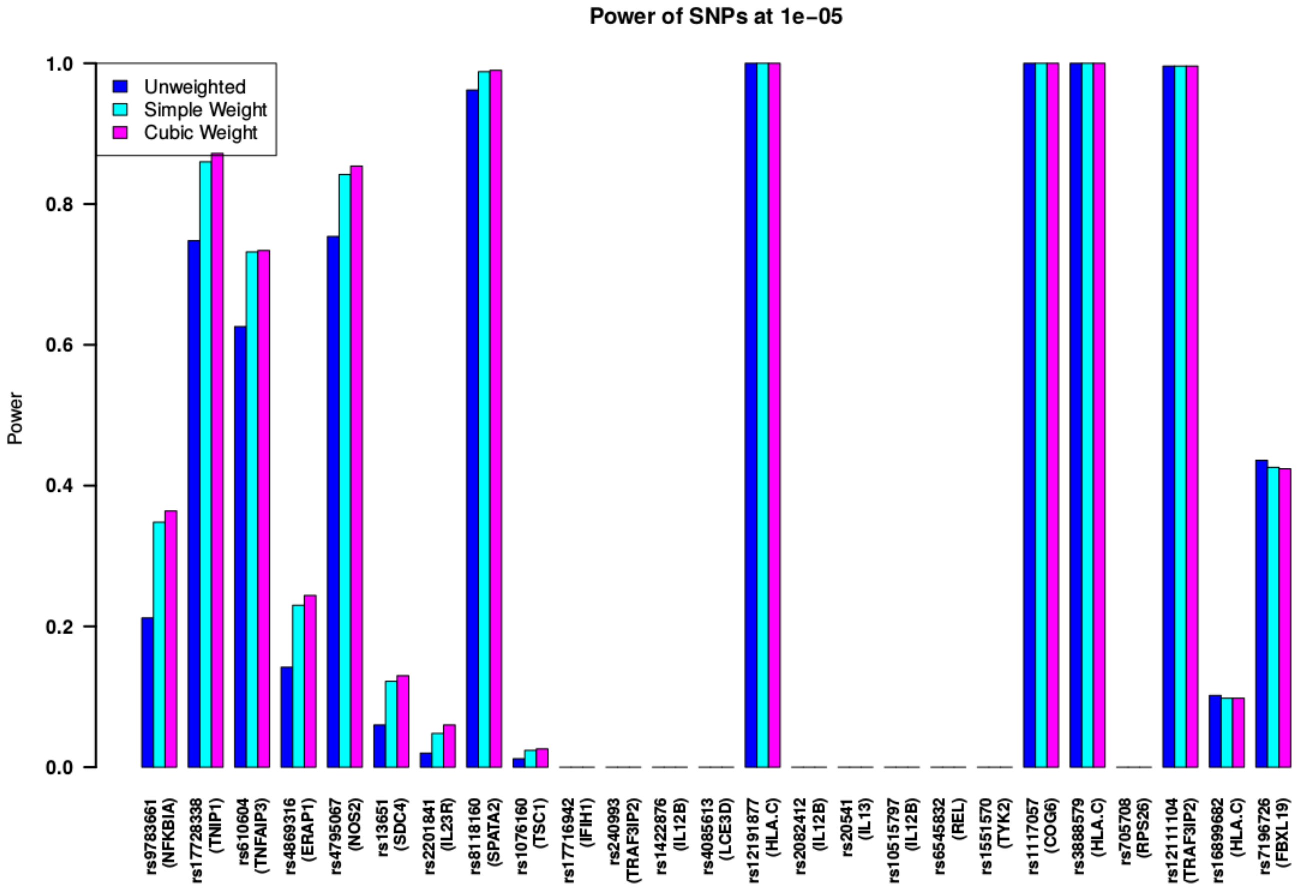

Figure S2: Barplot showing power to detect each causal SNP for different weighting schemes. X-axis shows the 25 causal SNPs and mapping genes and Y-axis shows the power based on 500 simulations. The bars filled with blue, cyan and pink stand for unweighted analysis, simple-weighting (SPW) and cubic weighting (CPW) respectively.

association results from some independent studies are summarized in **Table** S1.

S2.2.1 Re-weighted GWAS of Psoriasis

We obtained summary results for the psoriasis data on 1677 subjects downloaded from dbGAP (analysis described above). Cross validation (10-fold) selected 61 DFs (pathways). Only 2 loci (HLA-C on chromosome 6 and IL12B on chromosome 5) were found to be significant. All the annotations showed the SNP rs20541 on 5q31 to be genome-wide significant (GWS) i.e., weighted P-values < 5e-08, while the original p-value was 5.98e-07. This SNP did not reach genome-wide significance (GWS) in the discovery phase of the published GWAS which had a considerably larger sample size (comprising 1409 cases and 1436 controls). It was however GWS in the much larger validation stage consisting of 15,369 cases and 19,517 controls (p=5e-09). It has also been found to be GWS in other independent studies (Yin et al., 2015). **Figure** S4 shows the

Table S1: Results of re-weighted analysis of Psoriasis GWAS data for 18 known psoriasis associated SNPs using 4 different annotation sets.

SNPs	CHR	Gene	Original Study Summary Results			Weighted Pvalue	Previous Report	
			OR	CI	Unweighted Pvalue		Reported Pvalue	Reference
**Psoriasis GWAS**								
rs20541	5q31	IL13	1.14	1.09-1.19	$5.98E^{-07}$	$4.78E^{-09}$	$5.00E^{-09}$	Yin et.al
**SLE GWAS**								
rs13277113	8p23	BLK	1.39	1.26-1.54	$7.57E^{-08}$	$2.6E^{-09}$	$2.00E^{-10}$	Lee et.al
rs4548893	16p11	ITGAM/ ITGAX	1.28	1.16–1.43	$1.39E^{-06}$	$5.35E^{-09}$	$2.36E^{-12}$	Jacob et.al/Hom et.al
**CVD GWAS**								
rs840616	2q32	TFPI	0.92	0.89-0.96	$4.09E^{-06}$	$2.06E^{-08}$	$7.37E^{-07}$	C4D Consortium
rs762551	15q24	CYP2A1	0.926	0.895-0.96	$8.13E^{-06}$	$2.25E^{-08}$	$4.00E^{-02}$	Cornelis et.al
rs6504218	17q23	PECAM1	0.924	0.894-0.956	$5.54E^{-06}$	$5.21E-^{008}$	$4.92E^{-28}$	Howson et.al
rs10160170	10q11	CXCL12	1.127	1.077-1.179	$4.15E^{-06}$	$3.38E - 008$	$2.12E^{-10}$	Schunkert et.al
**Type II Diabetes GWAS**								
rs223340	4q24	UBE2D3	1.14	0.08–0.21	$3.90E^{-06}$	$2.56E^{-08}$	$4.79E^{-05}$	Christian et.al
rs231362	11p15	KCNQ1	1.11	1.07-1.16	$1.40E^{-07}$	$3.36E^{-008}$	$2.80E^{-13}$	Voight et.al
rs11603334	11q13	ARAP1	1.13	01.08-1.19	$5.50E^{-07}$	$4.90E^{-008}$	$3.30E^{-38}$	Strawbridge et.al
rs2612069	12q14	HMGA2	1.16	1.10-1.23	$7.70E^{-08}$	$5.99E^{-009}$	$2.75E^{-12}$	Ng.et.al
rs11020107	12q24	HNF1A	1.08	1.05-1.12	$1.0E^{-06}$	$4.59E^{-008}$	$7.80E^{-08}$	Morris et.al
rs4420638	19q13	APOC1	0.08	0.05-0.104	$3.20E^{-07}$	$4.06E^{-008}$	$9E^{-08}$	Zhao et.al

Manhattan plots of p-values before and after weighting (with 'Merged' annotation set). As seen from the Manhattan, in this case the IL13 locus is the only locus that can be considered as a 'crossover' locus in the sense that in this region 'no SNP was GWS' before weighting while one or more SNPs became GWS after weighting.

S2.2.2 GWAS of SLE (Systemic Lupus Erythematosus)

We downloaded summary data on SLE from the International Consortium on the Genetics of Systemic Lupus Erythematosus (SLEGEN) (Harley et al., 2008) from dbGAP. These summary data were based on 767 women with SLE and 383 control women available from dbGAP. Unweighted analysis showed 3 GWS loci. Cross validation selected 840 DFs (pathways). Pathway guided GWAS with the 'Merged' annotation set gave 2 additional 'crossover' GWS loci; one near the BLK gene on chromosome 8p23 and another near the genes ITGAM and ITGAX on chromosome 16p11. The Manhattan plots before and after weighting are showed in **Figure** S5. The lead SNP in the BLK locus was rs13277113 that has been identified as associated with SLE from other studies (Hom et al., 2008). Similarly, rs4548893 a crossover SNP in the ITGAM locus has been reported previously as GWS (Harley et al., 2008; Hom et al., 2008).

S2.2.3 GWAS of Coronary Artery Disease (CAD):

Summary data on Coronary Artery Disease was downloaded from CARDIoGRAMplusC4D consortium. The data is based on study involving 15,420 cases and 15,062 controls from European and South Asian population (Coronary Artery Disease (C4D) Genetics Consortium, 2011). Unweighted analysis showed 15 GWA significant loci. Pathway guided GWAS with the 'Merged' annotation set on cross validation selected 902 DFs (pathways) and gave 4 'crossover' GWAS loci. The Manhattan plots before and after weighting are showed in **Figure** S6. One crossover locus on 17q23 (lead SNP rs6504218) is near the PECAM1 gene. This region has been identified as a novel locus (lead SNP rs1867624) recently in a larger GWAS meta-analysis comprising 88192 cases and 162544 controls (Howson et al., 2017). Interestingly these two SNPs (rs6504218 and rs1867624) are in strong LD and the former SNP is an eQTL for PECAM1 in aortic endothelial cells (Howson et al., 2017). The previously reported SNP rs1867624 was not available in our data. Another crossover locus was on 10q11 near gene CXCL12 (lead SNP rs10160170). SNPs within this region (e.g. rs1746048 less than 300 kb from rs10160170) have been confirmed to be associated with CAD previously (Schunkert et al., 2011). The crossover locus on 15q24 is near CYP1A2 gene (lead SNP rs762551). This SNP codes for the CYP1A2*1F allele (164A>C) of the CYP1A2 gene. The 'C' allele is known to be a slower metabolizer of caffeine and has potential interaction with coffee intake in CAD (Cornelis, El-Sohemy, Kabagambe, & Campos, 2006) and several other phenotypes (e.g., hypertension, Parkinson's disease, breast cancer etc). However, the marginal association of this allele with CAD (as found here) could be due to residual confounding with other factors such as age, sex and smoking. The fourth crossover locus was near the TFPI gene on 2q32 (lead SNP rs840616). This SNP was reported as suggestive association for the study we analysed (Coronary Artery Disease (C4D) Genetics Consortium, 2011), but not found in subsequent larger meta-analyses. While it is possible that it is a false positive, it could also have a population specific effect or an interaction with environmental factor(s). Interestingly, the expression of TFPI (Tissue Factor Pathway Inhibitor) has been linked to risk of thrombosis and heart disease previously (Falciani et al., 1998) and a coding SNP in this region (rs7586970 or TFPI N221S within 500 KB) has been shown to be associated with total plasma TFPI levels (Dennis, 2016).

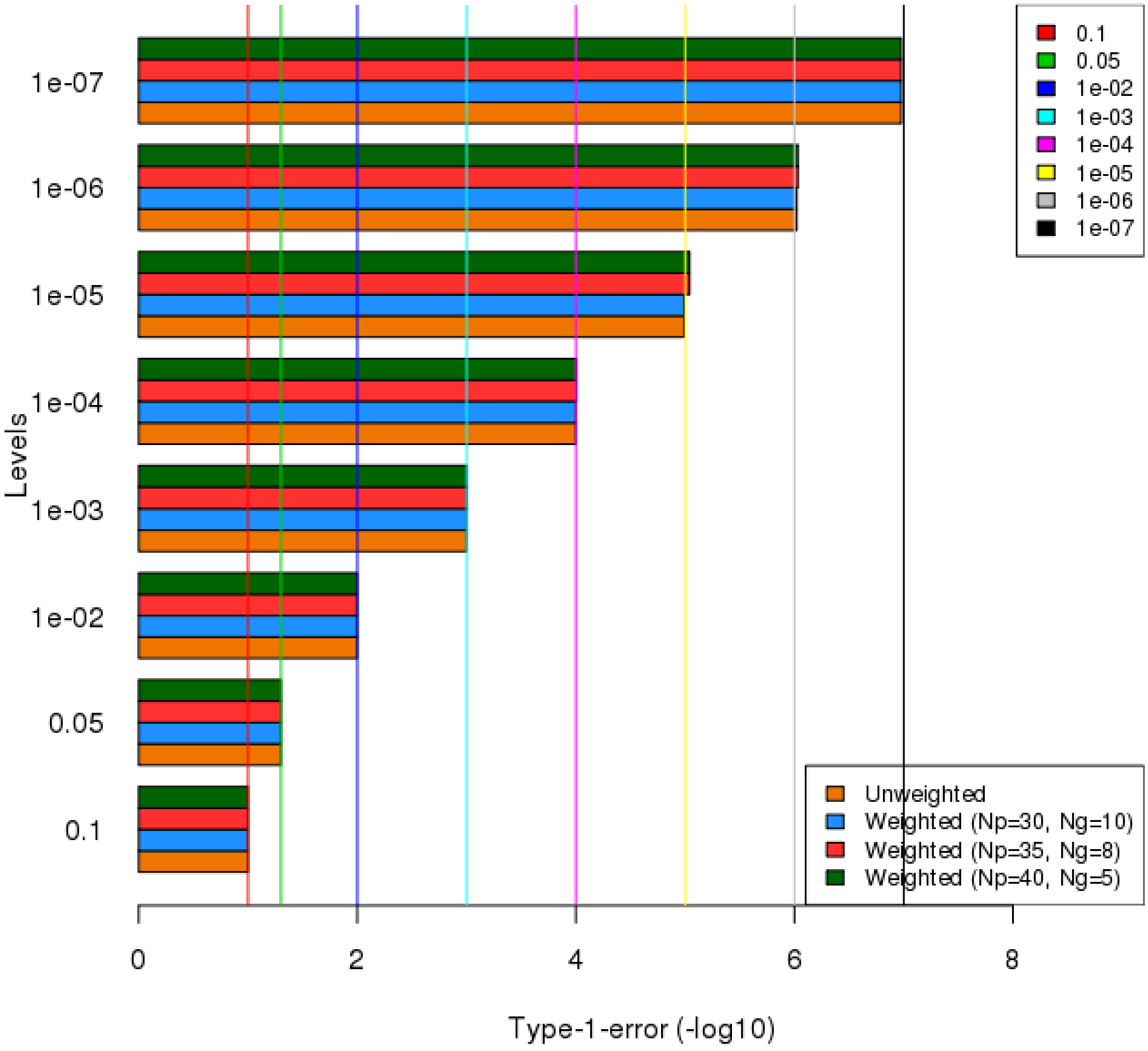

Figure S3: Barplot of type-1 error with varying levels of connectivity between genes using synthetic pathway lists. Here N_p denotes number of pathways (selected to be enriched by 'null genes') and N_g denotes number of 'null genes' allocated to each such pathway. X-axis shows -log10 values of type-1 error achieved and Y-axis shows the significance level at which each SNP is rejected (target level). Green, red, blue and orange bars show global type-1 error (-log10 scale) across significance levels respectively for unweighted analysis, first synthetic pathway list (T_p = 40, T_g = 5 i.e. low connectivity), second pathway list (T_p = 35, T_g = 8 i.e. moderate connectivity) and third pathway list (T_p = 30, T_g = 10 i.e. high connectivity among null genes)

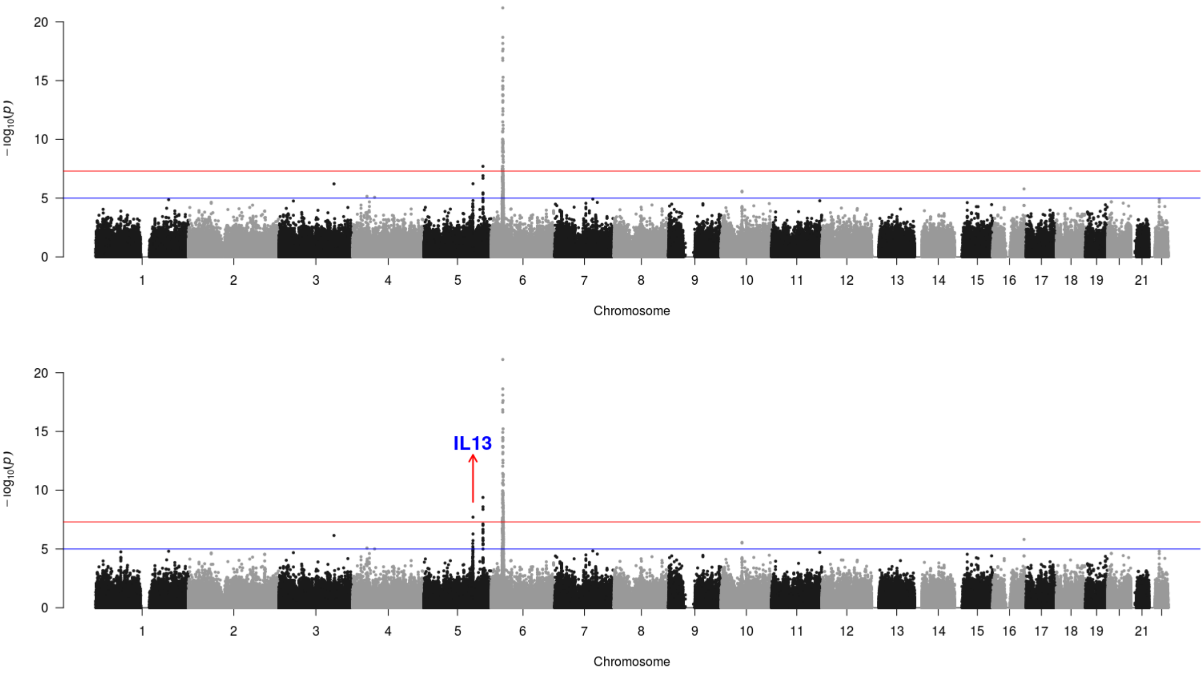

Figure S4: Manhattan plots of Psoriasis GWAS before and after weighted analysis. Upper and lower panels denote Manhattans of unweighted p-values and p-values weighted by 'Merged' annotation set (with cubic weighting). IL13 locus is shown as the only 'crossover' region (i.e. region newly detected by weighted analysis).

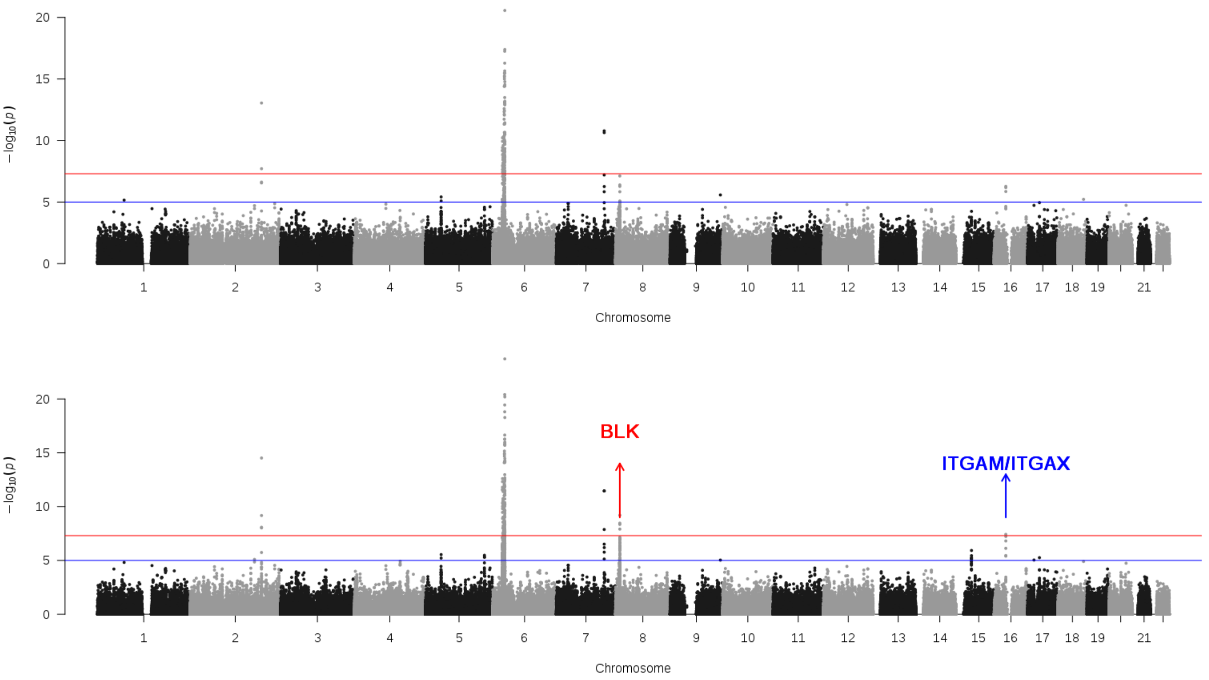

Figure S5: Manhattan plots of Psoriasis SLE (Lupus) before and after weighted analysis. Upper and lower panels denote Manhattans of unweighted p-values and p-values weighted by 'Merged' annotation set (with cubic weighting). Two crossover loci (i.e. region newly detected by weighted analysis) are shown with names of genes mapping to those regions.

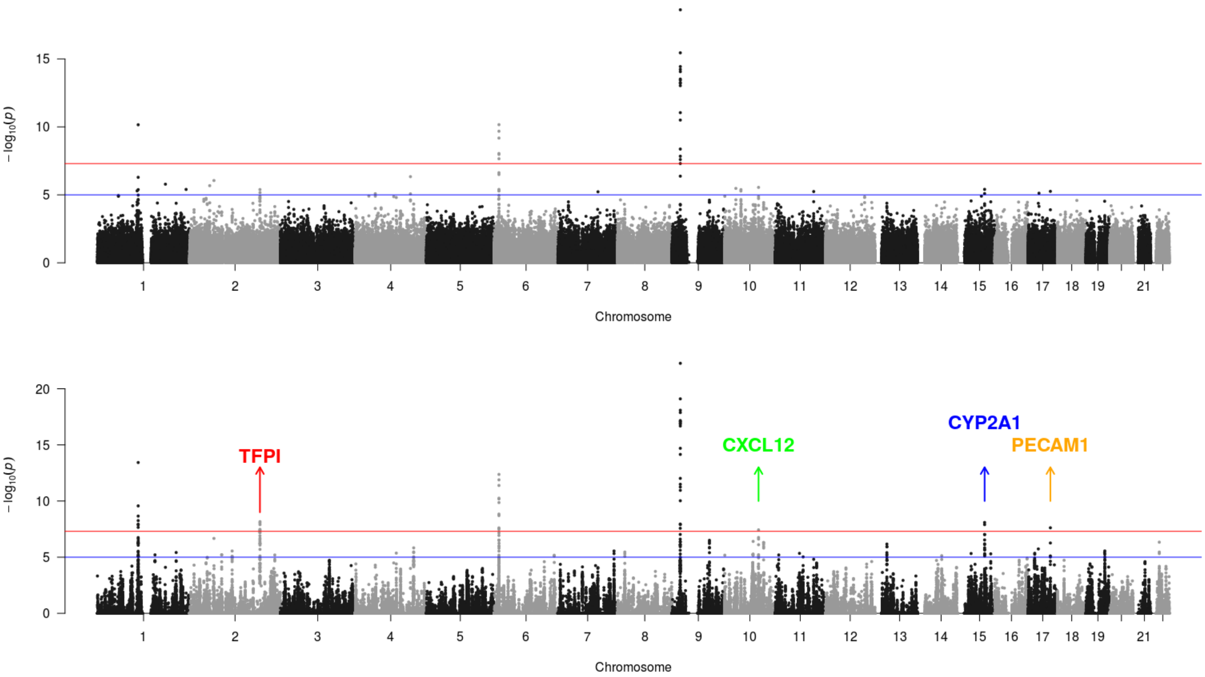

Figure S6: Manhattan plots of Psoriasis Coronary Artery Disease (CAD) before and after weighted analysis. Upper and lower panels denote Manhattans of unweighted p-values and p-values weighted by 'Merged' annotation set (with cubic weighting). Four crossover loci (i.e. region newly detected by weighted analysis) are shown with names of genes mapping to those regions.

S3 Data Sources

The data/summaries used for the analyses of Psoriasis described in this manuscript were obtained from the database of Genotypes and Phenotypes (dbGaP) found at http://www.ncbi.nlm.nih.gov/gap through dbGaP accession number phs000019. Funding for the Collaborative Association Study of Psoriasis was provided by the National Institutes of Health, the Foundation for the National Institutes of Health, and the National Psoriasis Foundation. Support for genotyping of samples was provided through the Genetic Association Information Network (GAIN). Samples and associated phenotype data for the Collaborative Association Study of Psoriasis were provided by Drs. James T Elder (University of Michigan, Ann Arbor, MI), Gerald G Krueger (University of Utah, Salt Lake City, UT), Anne Bowcock (Washington University, St. Louis, MO) and Gonçalo R Abecasis (University of Michigan, Ann Arbor, MI). For a description of the dataset, phenotypes, genotype data and quality control procedures see Nair et al (2009) Nature Genetics 41:200-204. The summary results used for the analyses of SLE described in this article were obtained from the database of Genotypes and Phenotypes (dbGaP), at http://www.ncbi.nlm.nih.gov/gap. Genotype and phenotype data for the International Consortium on the Genetics of Systemic Lupus Erythematosus (SLEGEN) (dbGaP accession number phs000216.v1.p1) were provided by Carl D. Langefeld. Funding support for the original study was provided by the Alliance for Lupus Research, the National Institutes of Health, and other sources as detailed in International Consortium for Systemic Lupus Erythematosus Genetics (SLEGEN), Harley JB, Alarcón-Riquelme ME, Criswell LA, Jacob CO, Kimberly RP, Moser KL, Tsao BP, Vyse TJ, Langefeld CD. Genome-wide association scan in women with systemic lupus erythematosus identifies susceptibility variants in ITGAM, PXK, KIAA1542 and other loci. Nat Genet. 2008. 40(2):204-10.

References

Ashburner, M., Ball, C. A., Blake, J. A., Botstein, D., Butler, H., Cherry, J. M., . . . Sherlock, G. (2000, May). Gene ontology: tool for the unification of biology. The Gene Ontology Consortium. *Nature Genetics*, *25*(1), 25–29. doi: 10.1038/75556

Carlson, M. (2016). *KEGG.db: A set of annotation maps for KEGG*.

Carlson, M. (2017a). *GO.db: A set of annotation maps describing the entire Gene Ontology*.

Carlson, M. (2017b). *org.Hs.eg.db: Genome wide annotation for Human*.

Cornelis, M. C., El-Sohemy, A., Kabagambe, E. K., & Campos, H. (2006). Coffee, CYP1a2 genotype, and risk of myocardial infarction. *Jama*, *295*(10), 1135–1141.

Coronary Artery Disease (C4D) Genetics Consortium. (2011, March). A genome-wide association study in Europeans and South Asians identifies five new loci for coronary artery disease. *Nature Genetics*, *43*(4), 339–344. doi: 10.1038/ng.782

Csardi, G., & Nepusz, T. (2006). The igraph software package for complex network research. *InterJournal*, *Complex Systems*, 1695. Retrieved from `http://igraph.org`

Dennis, J. (2016). *Genetic and Epigenetic Determinants of Tissue Factor Pathway Inhibitor Plasma Levels* (Unpublished doctoral dissertation). University of Toronto (Canada).

Falciani, M., Gori, A. M., Fedi, S., Chiarugi, L., Simonetti, I., Dabizzi, R. P., . . . others (1998). Elevated tissue factor and tissue factor pathway inhibitor circulating levels in ischaemic heart disease patients. *Thrombosis and haemostasis*, *79*(3), 495–499.

Harley, J. B., Alarcón-Riquelme, M. E., Criswell, L. A., Jacob, C. O., Kimberly, R. P., Moser, K. L., . . . Langefeld, C. D. (2008). Genome-wide association scan in women with systemic lupus erythematosus identifies susceptibility variants in ITGAM, PXK, KIAA1542 and other loci. *Nature genetics*, *40*(2), 204–210.

Hom, G., Graham, R. R., Modrek, B., Taylor, K. E., Ortmann, W., Garnier, S., . . . others (2008). Association of systemic lupus erythematosus with C8orf13–BLK and ITGAM–ITGAX. *New England Journal of Medicine*, *358*(9), 900–909.

Howson, J. M. M., Zhao, W., Barnes, D. R., Ho, W.-K., Young, R., Paul, D. S., . . . Saleheen, D. (2017, July). Fifteen new risk loci for coronary artery disease highlight arterial-wall-specific mechanisms. *Nature Genetics*, *49*(7), 1113–1119. doi: 10.1038/ng.3874

Kanehisa, M., & Goto, S. (2000). KEGG: kyoto encyclopedia of genes and genomes. *Nucleic acids research*, *28*(1), 27–30.

Matys, V., Kel-Margoulis, O. V., Fricke, E., Liebich, I., Land, S., Barre-Dirrie, A., . . . Wingender, E. (2006, January). TRANSFAC and its module TRANSCompel: transcriptional gene regulation in eukaryotes. *Nucleic Acids Research*, *34*(Database issue), D108–110. doi: 10.1093/nar/gkj143

Price, A. L., Patterson, N. J., Plenge, R. M., Weinblatt, M. E., Shadick, N. A., & Reich, D. (2006). Principal components analysis corrects for stratification in genome-wide association studies. *Nature genetics*, *38*(8), 904–909.

Roeder, K., Devlin, B., & Wasserman, L. (2007). Improving power in genome-wide association studies: weights tip the scale. *Genet Epidemiol*, *31*(7), 741–7.

Schunkert, H., König, I. R., Kathiresan, S., Reilly, M. P., Assimes, T. L., Holm, H., . . . Samani, N. J. (2011, March). Large-scale association analysis identifies 13 new susceptibility loci for coronary artery disease.

Nature Genetics, *43*(4), 333–338. doi: 10.1038/ng.784
Tryka, K. A., Hao, L., Sturcke, A., Jin, Y., Wang, Z. Y., Ziyabari, L., . . . others (2013). NCBI's Database of Genotypes and Phenotypes: dbGaP. *Nucleic acids research*, *42*(D1), D975–D979.
Yin, X., Low, H. Q., Wang, L., Li, Y., Ellinghaus, E., Han, J., . . . Liu, J. (2015, April). Genome-wide meta-analysis identifies multiple novel associations and ethnic heterogeneity of psoriasis susceptibility. *Nature Communications*, *6*, 6916. doi: 10.1038/ncomms7916
